# *Mycobacterium avium-intracellulare* complex promote release of pro-inflammatory enzymes matrix metalloproteinases by inducing neutrophil extracellular trap formation

**DOI:** 10.1038/s41598-022-09017-y

**Published:** 2022-04-11

**Authors:** Kota Nakamura, Hitoshi Nakayama, Shinichi Sasaki, Kazuhisa Takahashi, Kazuhisa Iwabuchi

**Affiliations:** 1 Department of Respiratory Medicine, Juntendo University Faculty of Medicine, Bunkyo-ku, Tokyo, Japan; 2 Laboratory of Biochemistry, Juntendo University Faculty of Health Care and Nursing, Urayasu, Chiba Japan; 3 Institute for Environmental and Gender-Specific Medicine, Juntendo University Graduate School of Medicine, Urayasu, Chiba Japan; 4 Infection Control Nursing, Juntendo University Graduate School of Health Care and Nursing, Urayasu, Chiba Japan

**Keywords:** Infection, Mechanisms of disease

## Abstract

The prevalence of and mortality from non-tuberculous mycobacteria (NTM) infections have been steadily increasing worldwide. Most NTM infections are caused by *Mycobacterium avium-intracellulare* complex (MAC). MAC can escape from killing by neutrophils, which are professional phagocytes. However, the involvement of neutrophils in the pathogenesis of MAC infection is poorly understood. The present study assessed the roles of neutrophil extracellular trap (NET) formation in neutrophil defense mechanisms against infection with MAC strains, including *M. avium* isolated from patients with severe or mild lung tissue destruction. Although all MAC induced NET formation, non-pathogenic mycobacteria (*M. gordonae* and *M. smegmatis*) slightly but not significantly induced NET formation. Peptidylarginine deiminase 4 (PAD4) inhibitor reduced MAC-induced NET formation but did not affect MAC escape from neutrophils. PAD4 inhibition attenuated the MAC-induced matrix metalloproteinase (MMP)-8 and 9 release to the levels of MMPs from non-pathogenic mycobacteria. MAC also induced interleukin (IL)-8 release by neutrophils, a process independent of MAC-induced NET formation. Taken together, these findings suggest that MAC induce NET formation, IL-8 release and NETs-dependent release of MMP-8 and -9 from neutrophils, leading to neutrophil accumulation and further inflammation, thereby enhancing the progression of infection in the lungs.

## Introduction

In recent years, the prevalence of^1,2^ and mortality from^3,4^ non-tuberculous mycobacteria (NTM) infections have increased worldwide, emphasizing the importance of determining the mechanisms of lung tissue destruction in patients with NTM infection. Most NTM infections are caused by *Mycobacterium avium-intracellulare* complex (MAC). These bacteria are distributed throughout a broad range of environments, including water, soil, and dust particles^5^. MAC mainly infects the lungs and induces long-term inflammation^5^, involving pathological changes to lung tissue, such as granulation, cavitation, and bronchiectasis^6,7^, and leading to lung tissue destruction. For example, exacerbated lung tissue destruction and CT images showing cavitation and bronchiectasis have been observed in MAC-infected patients^8,9^. The possibility of rapid lung destruction in some MAC patients has led to the initiation of immediate antimicrobial treatment for patients with pulmonary cavities or extensive disease^10,11^. In contrast, some MAC patients do not show lung destruction, even without antimicrobial treatments^12^. These findings suggest that host factors and/or susceptibility to MAC can affect the progression of lung tissue destruction.

Neutrophils are the most abundant leukocytes in blood and play a central role in innate immune responses against mycobacterial infection^13,14^. These cells are immediately recruited to the site of injury or infection through chemokines such as interleukin-8 (IL-8) and leukotriene B_4_^15^. Neutrophils kill pathogens via several major pathways, including phagocytosis, superoxide generation, degranulation and the release of neutrophil extracellular traps (NETs)^16^. NETs manifest as large, extracellular, web-like structures of chromatin filaments composed of histones, proteases, and granule proteins^17^. In addition to microorganisms, proinflammatory host molecules, such as IL-8, induce NET formation^16,18^. NET is thought to prevent microbial dissemination by trapping them in DNA web-like structures and killing them through antimicrobial molecules^17^. NET-associated molecules, however, may induce uncontrolled inflammatory responses causing tissue damage^16^. Although in vitro studies using human neutrophils^19^, as well as studies using human lung pathological samples^20^, blood samples^21^ and sputum^22^ have shown that *M. tuberculosis* induces NET formation, little is known about MAC-induced NET formation.

Interstitial collagen is the most abundant macromolecule constituent of the large airways, blood vessels, and alveolar interstitium in the lungs^23^. Lung tissue destruction requires the destruction of such collagen. Matrix metalloproteinases (MMPs) are a family of zinc-dependent endoproteinases that degrade various types of collagen^24^. In human neutrophils, MMP-8 and MMP-9 are stored as latent enzymes (pro-MMP-8 and pro-MMP-9) within specific granules and gelatinase granules, respectively^25^. MMPs play an important role in many biological processes, including cell proliferation, migration, and differentiation, remodeling of the extracellular matrix, and tissue invasion and vascularization. These biological processes take place multiple times in vivo. However, if not balanced, MMPs can also contribute to harmful pathological conditions^24^, including the pathogenesis of several respiratory diseases, such as idiopathic pulmonary fibrosis^26^, emphysema^27^ and pulmonary tuberculosis^28^.

The present study was designed to assess the relationship between MAC pathogenicity and NET formation by examining MAC-induced NET formation and the anti-mycobacterial activity of NETs. Furthermore, mechanisms underlying the release of neutrophil granules and cytokine production during MAC-induced NET formation were investigated.

## Materials and methods

### Reagents and antibodies

Alexa Fluor 633-protein labeling kits (A20170), TO-PRO3 (T3605), Sytox orange (S11368), 3, 3', 5, 5'-tetramethylbenzidine (TMB) and sulfuric acid for use with the TMB substrate were from Invitrogen (Thermo Fisher Scientific, Waltham, MA, USA). Antibodies to human citrullinated histone H3 (cit-H3; ab5103) and myeloperoxidase (MPO; ab25989), Alexa Fluor 488 (ab150077), Alexa Fluor 568 (ab175473), and GSK106 (ab229184) were from abcam (Cambridge, UK). Antibody to human H3 (#4499) was from Cell Signaling Technology (Danvers, MA, USA); antibody to human β-actin antibody was from Sigma-Aldrich (St. Louis, MO, USA), and A23187 (11016) and GSK484 (17488) were from Cayman (Ann Arbor, MI, USA).

### Isolation of human neutrophils

Ethical approval for obtaining blood from healthy human volunteers was provided by the Ethics Review Board of Juntendo University Faculty of Medicine (Authorization number: 2019073). All research was performed in accordance with relevant guidelines/regulations, and written informed consent was obtained from all participants.

Human neutrophils were isolated from heparinized peripheral blood of healthy volunteer subjects by Polymorphprep™ (Nycomed Pharma; Oslo, Norway) centrifugation. Neutrophil populations were > 95% pure as determined by Turk's solution staining (FUJIFILM Wako Pure Chemical Corporation; Osaka, Japan).

### Bacterial strains and culture conditions

MAC were obtained from stocks clinically isolated from non-HIV-infected patients at Juntendo University Hospital and Juntendo University Urayasu Hospital and identified as *M. intracellulare* and *M. avium* by DNA-DNA hybridization methods. The severity of lung tissue destruction was evaluated using a previously described scoring system^29^. Mycobacteria isolated from patients with severe lung tissue destruction and a critical clinical course was defined *M. avium* s whereas mycobacteria isolated from patients with mild lung tissue destruction and a favorable clinical course was define *M. avium* m. *M. gordonae* GTC 612 was from Gifu University, Japan. Mycobacteria were cultured in Middlebrook 7H9 broth (Difco Laboratories, BD), supplemented with OADC (Becton Dickinson).

### Labeling of mycobacteria with Alexa Fluor dye

Mycobacteria were suspended in phosphate-buffered saline (PBS). To each suspension was added a 100 μl aliquot of 1.0 M sodium hydrogen carbonate (NaHCO_3_), followed by incubation with Alexa Fluor 633 dye for 2 h at room temperature (RT) and extensive washing with PBS.

### Examination of NETs by confocal microscopy

Neutrophils, seeded on 0.01% poly L lysine (#0403, ScienCell) coated glass base dishes, were pre-incubated with the indicated inhibitors for 30 min at 37 °C, followed by incubation with mycobacteria at a multiplicity of infection (MOI) of 1 for 4 h at 37 °C under 5% CO_2_ atmosphere. PMA (20 nM) and A-23187 (1 µM) were used as pharmacological inducers of NET formation. The cells were subsequently fixed with 2% paraformaldehyde, permeabilized with 0.5% Triton X-100, and incubated for 2 h with 2% bovine serum albumin and 5% normal goat serum to prevent nonspecific binding. Then cells were incubated with antibodies against cit-H3 (ab5103, Abcam) and MPO (ab25289, Abcam), followed by incubation with the secondary antibodies (Alexa Fluor 488 (ab150077, Abcam) and Alexa Fluor 568(ab175473, Abcam). DNA was stained with TO-PRO3 (T3605, Invitrogen) or SYTOX orange (S11368, Invitrogen). Cells and NET formation were monitored using a STED microscope Leica TCS SP5(Leica Microsystems GmbH, Wetzlar, Germany).

### Quantification of NET formation using confocal images

NET formation was quantitated using confocal images by determining the distribution of the nuclear area^30,31^ and the percentage of cit-H3 positive cells^32^. Neutrophils positive for cit-H3 were counted and divided by the number of TO-PRO3-positive neutrophils to determine the percentage of cit-H3 positive cells. Quantification by the distribution of nuclear area is based on changes in nuclear size and neutrophil shape during NET formation. TO-PRO3 images of nuclei were analyzed using ImageJ image processing software (US National Institutes of Health). Each sample consisted of 300 cells, and the TO-PRO3-positive area was individually measured in each sample. The distribution of the number of cells across the range of nuclear area was determined using the frequency function in Excel (Microsoft). The data were plotted as the percentage of cells positive for TO-PRO3 for each DNA area range (Supplementary Fig. 1). TO-PRO3-positive areas ranged from 30–150 µm^2^ for unstimulated neutrophils, with the positive areas for > 95.6% of cells being < 120 µm^2^. The TO-PRO3-positive areas of PMA- and A-23187-treated cells were expanded up to 350 µm^2^. Thus, NET formation was defined as cells with nuclear areas > 120 µm^2^.

### Cytokine/chemokine array

Neutrophils were pre-incubated in the presence or absence of GSK484 and stimulated with mycobacteria at a MOI of 1 for 4 h at 37 °C. The culture supernatants were collected and subjected to cytokine array analysis (ARY005B, R&D Systems, Minneapolis, MN, USA) according to manufacturer's instructions. Briefly, human cytokine array panel A membranes (R&D Systems) were incubated with cell culture supernatants. After washing, the membranes were further incubated with a detection Ab cocktail for 1 h. Spot detection was performed with streptavidin–horseradish peroxidase. The intensity of the spot was measured with ImageJ software (U.S. National Institutes of Health; http://rsb.info.nih.gov/ij/).

### ELISA for neutrophil granules and cytokine

The concentrations of MPO, MMPs and the cytokines IL-1β, IL-8, and tumor necrosis factor (TNF)-α in cell culture supernatants were measured using the DuoSet enzyme-linked immunosorbent assay (ELISA) development system (R&D Systems) and ELISA MAX Standard Set Human TNF-α (Cat# 430201; BioLegend, San Diego, CA, USA) according to manufacturer' instructions. The absorbance at 450 nm was measured on a microplate reader (2030 ARVO X4; PerkinElmer Japan; Tokyo).

### Western blotting

Samples were resolved by 14% SDS-PAGE and transferred to PVDF membranes. To evaluate the citrullination of histone H3 by mycobacteria, neutrophils were preincubated in the presence or absence of GSK484/109, then stimulated with mycobacteria at MOI of 1 for 4 h at 37 °C. After stimulation, the cells were lysed by Dounce homogenization in lysis buffer; and the proteins were resolved by SDS-PAGE; and subjected to Western blotting. The blots were incubated with primary antibody to citrullinated histone H3, followed by incubation with horseradish peroxidase–conjugated secondary antibodies. Membranes were stripped of antibodies by incubation with stripping buffer [62.5 mM Tris–HCl (pH 6.8), 100 mM β-mercaptoethanol, 2% SDS] for 30 min at 55 °C, followed by incubation with antibodies against total histone H3 and β-actin. Bands detected with SuperSignal reagent (Pierce Chemical) were scanned, and chemiluminescence signal intensities were quantified with ImageJ software (U.S. National Institutes of Health; http://rsb.info.nih.gov/ij/). The extent of histone H3 citrullination was calculated as the ratio of the band intensity of citrullinated histone H3 to that of β-actin.

### Killing assay

Neutrophils (2 × 10^4^ cells in 100 μl DMEM/F12) were pre-incubated with indicated inhibitors for 30 min, followed by treatment with mycobacteria at an MOI of 1 in 96-well flat-bottom tissue culture plates (Asahi Techno Glass, Tokyo, Japan) for 24 h at 37 °C. The culture plates were treated with 0.1% Triton X-100-containing PBS for 5 min at RT. Neutrophil lysates were serially diluted tenfold in 0.05% Tween 80-containing PBS and plated on sterile 7H11 Middlebrook agar plates (Difco, BD). Colony forming units (cfu) were counted after 2 weeks of culture at 37 °C. Killing was calculated as the number of these mycobacterial colonies divided by the number after incubation in medium alone.

### Statistical analysis

Data were expressed as mean ± SD and analyzed by one-way analysis of variance (ANOVA), followed by Bonferroni’s multiple comparison test with GraphPad Prism software program (San Diego, CA). Differences between means were considered statistically significant at p < 0.05.

## Results

### MAC induce NET formation

**Figure 1:**
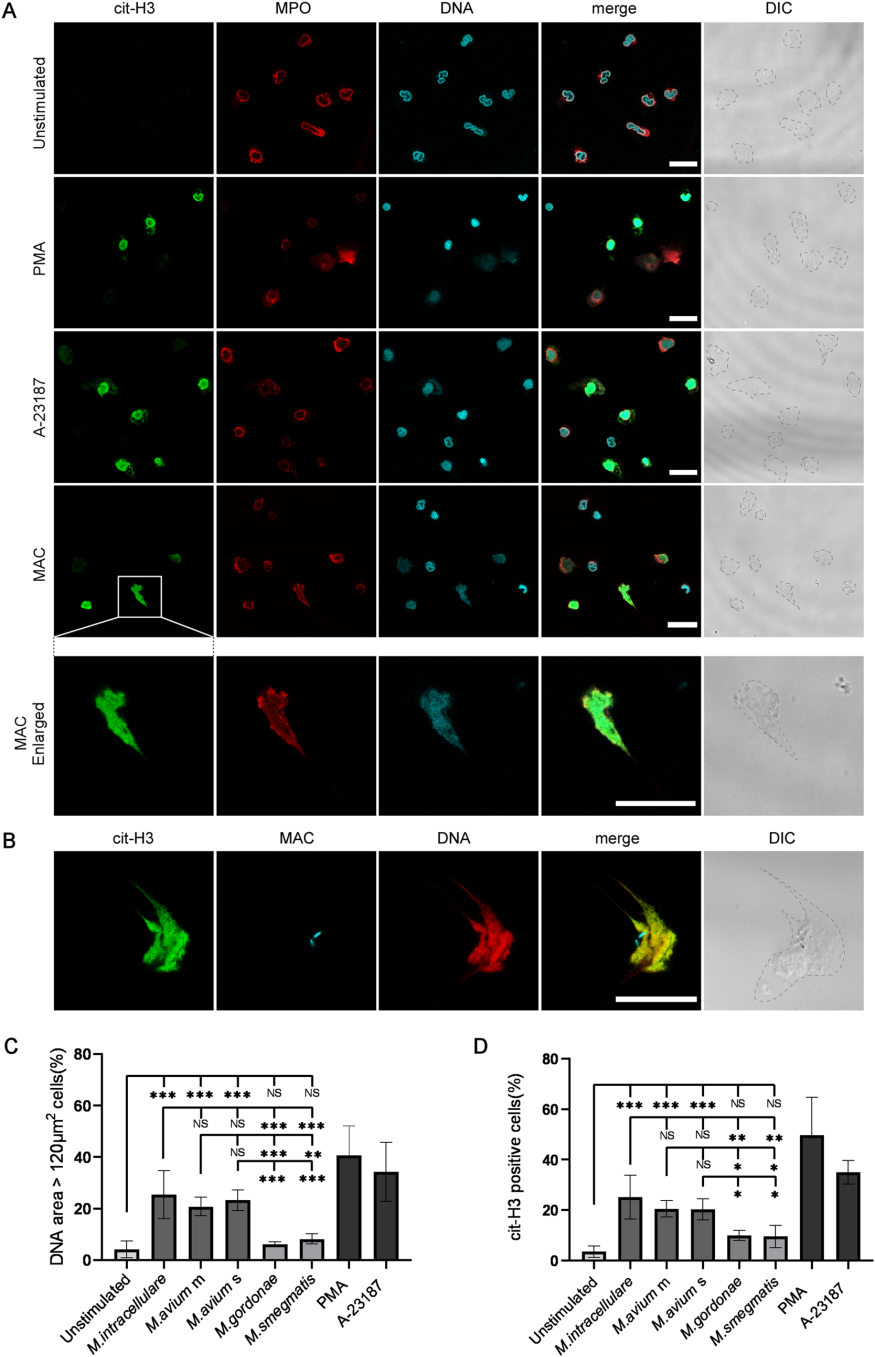
MAC induce NET formation. (**A**) Localization of NET-related molecules. Neutrophils were stained to identify nuclei (blue) and areas positive for cit-H3 (green) and MPO (red) to determine the structures of NETs. Neutrophils were treated with PMA and A-23187 as a positive control for NET formation. (**B**) Localization of *M. intracellulare* (MAC) and NET-related molecules. Neutrophils were treated with Alexa Fluor 633-conjugated antibody to *M. intracellulare* (blue) and stained to identify nuclei (red) and areas positive for cit-H3 (green). (**A**,**B**) Gray dotted lines in DIC images indicating the outline of neutrophils. Confocal images were acquired on a Leica TCS-SP5 confocal microscope equipped with a 1.4 numerical aperture (NA) × 63 objective lens. DIC; differential interference contrast. white scale bar; 25 μm. (**C**,**D**) Assessment of NET formation by DNA area expansion based on the percentage of cells with a nuclear area > 120 µm^2^ (**C**) and the percentage of cit-H3 positive neutrophils (**D**). *M. avium* m and s were the mycobacteria isolated from patients with mild lung tissue destruction and a favorable clinical course, and from patients with severe lung tissue destruction and a critical clinical course, respectively. We examined the experiments using *M. avium* m and s isolated from three different patients with triplicate, respectively. Data shown are the means ± standard deviations (SD) of three independent experiments. *P < 0.05, **P < 0.01, ***P < 0.001 by one-way analysis of variance (ANOVA) with Bonferroni's multiple comparison test.

Along with NET formation, histone citrullination has been found to promote chromatin decondensation, resulting in nuclear expansion^16^. To determine whether mycobacteria induce NET formation in human neutrophils, cells were stained with MPO, which adheres to NETs fibers^17^, to show the area occupied by NETs. The nuclei in unstimulated neutrophils had a typical lobulated shape, with few cit-H3-positive cells detected (Fig. 1A, Supplementary Fig. 2, 3). In contrast, the reagents PMA (a potent activator of PKC) and A-23187(a potent activator of PKC-ζ), which induce NET formation^33,34^, markedly reduced nuclear lobular structure, yielding many cit-H3 positive cells. Incubation with MAC induced cit-H3 positive neutrophils, with the cit-H3 positive area also stained with anti-MPO antibody (Fig. 1B, Supplementary Fig. 2, 3). These results suggest that MAC induce NET formation in human neutrophils.

Evaluation of pathogenic mycobacteria showed that *M. intracellulare*, *M. avium* m and *M. avium* s induced mean ± SD TO-PRO3 positive areas of 25.4 ± 9.4%, 20.8 ± 3.6% and 23.3 ± 4.0%, representing the percentages of neutrophils producing NETs (Fig. 1C, Supplementary Fig. 4). *M. gordonae* and *M. smegmatis*, which are generally considered nonpathogenic although they may cause disease in rare cases^35,36^, slightly but not significantly induced mean ± SD TO-PRO3 positive areas of 6.2 ± 1.0% and 8.3 ± 1.9%, respectively (Fig. 1C). Evaluation of citrullination, representing mycobacteria-induced NET formation by neutrophils (Fig. 1D, Supplementary Fig. 4), showed that *M. intracellulare*, *M. avium* m and *M. avium* s induced NET formation by 25.2 ± 8.8%, 20.5 ± 3.2% and 20.3 ± 4.2%, respectively, of neutrophils, whereas *M. gordonae* and *M. smegmatis* induced NET formation by 10.0 ± 2.1% and 9.6 ± 4.4%, respectively, of neutrophils. Both of these quantification methods revealed that, compared with non-pathogenic mycobacteria, MAC significantly promoted NET formation by neutrophils.

### PAD4 mediates MAC-induced NET formation

**Figure 2:**
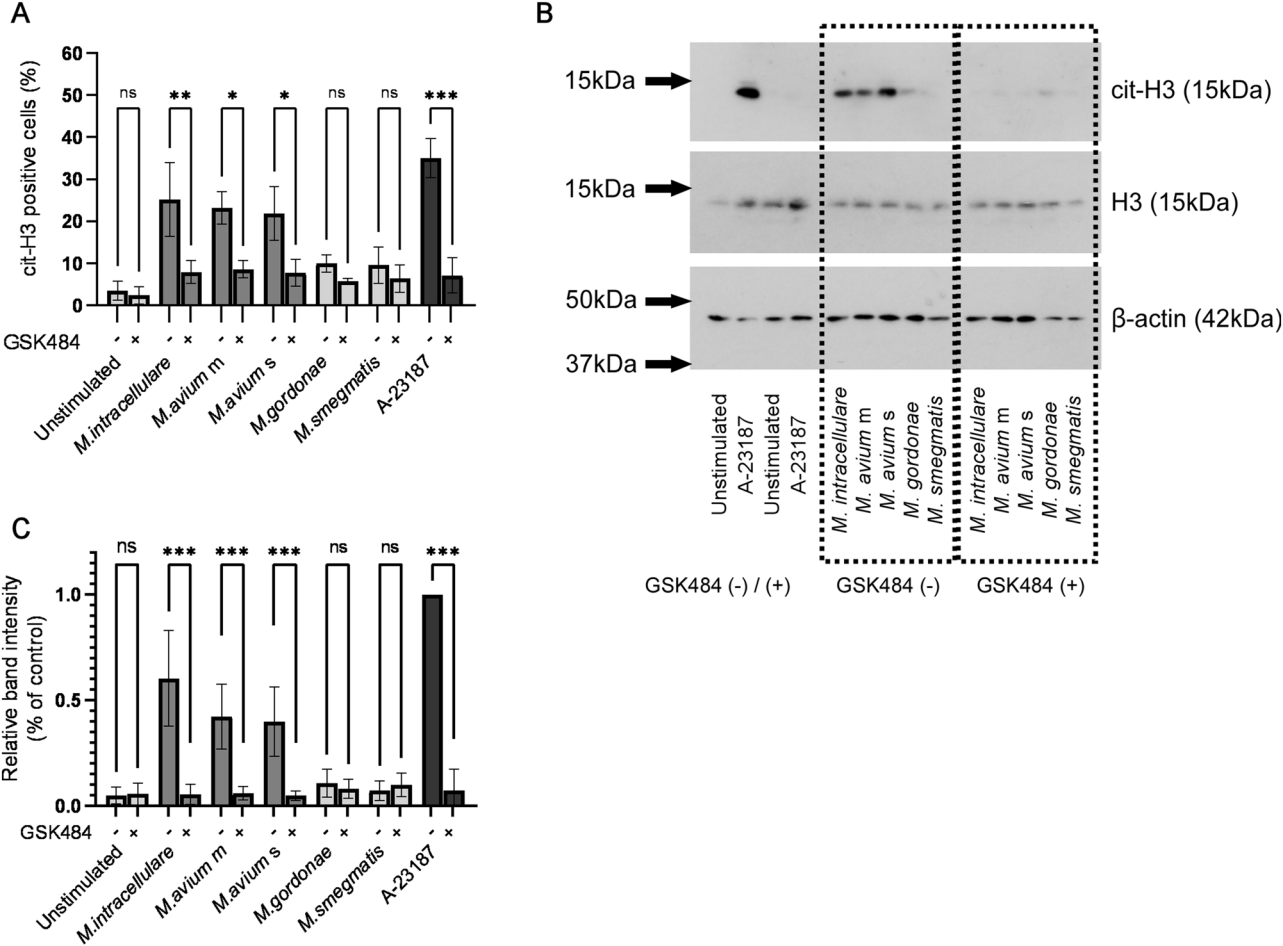
PAD4 mediates MAC-induced NET formation. (**A**) The effect of PAD4 inhibitor on the number of cit-H3 positive neutrophils. Data are means ± SD from five independent experiments. (**B**) The effect of PAD4 inhibitor on the expression levels of cit-H3. Representative protein band are shown. Molecular weights (kDa) are indicated on the left. H3 and β-actin were used as an internal control. Blots were cropped from different parts of the same gel. Original blots are presented in Supplementary Fig. 6. (**C**) Relative band intensities were calculated on the basis of densitometric analysis using ImageJ processing software. Protein levels of cit-H3 were calculated as the ratio of A-23187 pre-incubated with DMSO. Data are means ± SD from three independent experiments. *P < 0.05, **P < 0.01, ***P < 0.001 by one-way analysis of variance (ANOVA) with Bonferroni's multiple comparison test.

Peptidylarginine deiminase 4 (PAD4), which converts arginine to citrulline in histones, is highly expressed in human neutrophils. Citrullination by PAD4 reduces the strong positive charge of histones, thereby weakening histone-DNA binding. This weakened interaction subsequently leads to chromatin decondensation and NET formation^37^. The PAD4 inhibitor GSK484 suppressed induction by MAC of cit-H3 positive neutrophils (Fig. 2A) but had little effect on the percentage of cit-H3 positive neutrophils induced by non-pathogenic mycobacteria. The effect of the PAD4 inhibitor was further evaluated by assessing intracellular cit-H3 protein levels by Western blotting. PAD4 inhibitor significantly decreased intracellular cit-H3 protein band intensity of MAC-treated neutrophils (Fig. 2B,C), whereas GSK106, a negative control of PAD4 inhibitor, had no effect on cit-H3 protein band intensity of neutrophils (Supplementary Fig. 5). These results suggest that MAC-induced NET formation is regulated by PAD4. Evaluation of non-pathogenic mycobacteria showed that neutrophils produced slight amounts of GSK484-inhibitable cit-H3, although these cells rarely formed NETs.

### NET formation is not involved in neutrophil killing of mycobacteria

**Figure 3:**
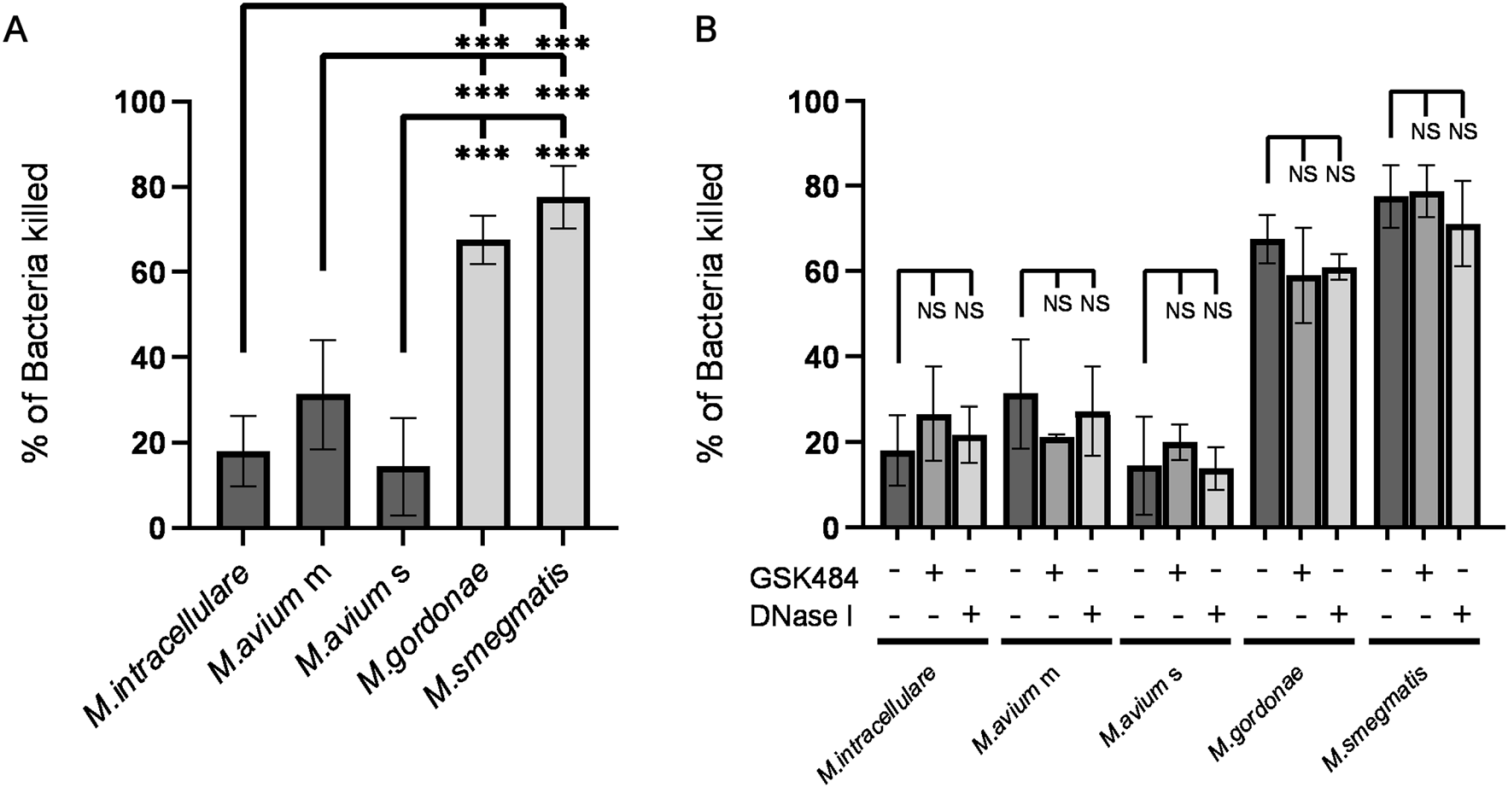
NET formation is not involved in neutrophil killing of mycobacteria. (**A**) Anti-mycobacterial activity of neutrophils. (**B**) Effect of inhibitors of NET formation on the anti-mycobacterial activity of neutrophils. Killing rate was calculated as the percentage of control values (bacteria incubated alone in the absence of neutrophils). Data are means ± SD of three independent experiments. *P < 0.05, **P < 0.01, ***P < 0.001 by one-way analysis of variance (ANOVA) with Bonferroni's multiple comparison test.

Because NET formation is thought to be involved in neutrophil killing of bacteria^17^, the present study assessed the involvement of NET formation in neutrophil killing of mycobacteria. In accordance with our previous experiments^38^, Neutrophils killed 67.5 ± 5.6% of *M. gordonae* and 77.5 ± 7.3% of *M. smegmatis*, but killed only 18.0 ± 8.2%, 31.3 ± 12.8% and 14.4 ± 11.5% of *M. intracellulare*, *M. avium* m and *M. avium* s, respectively (Fig. 3A), indicating that all MAC strains escaped from neutrophil killing. PAD4 inhibitor did not affect neutrophil killing of not only non-pathogenic mycobacteria but MAC (Fig. 3B). Although DNase I has been shown to inhibit NETs-dependent *S.aureus* killing by degrading NETs-associated DNA^39^, DNase I had little effect on neutrophil killing of any kind of mycobacteria. These results indicate that NET formation is not involved in neutrophil killing of mycobacteria.

### MAC-induced NET formation enhanced neutrophil degranulation

**Figure 4:**
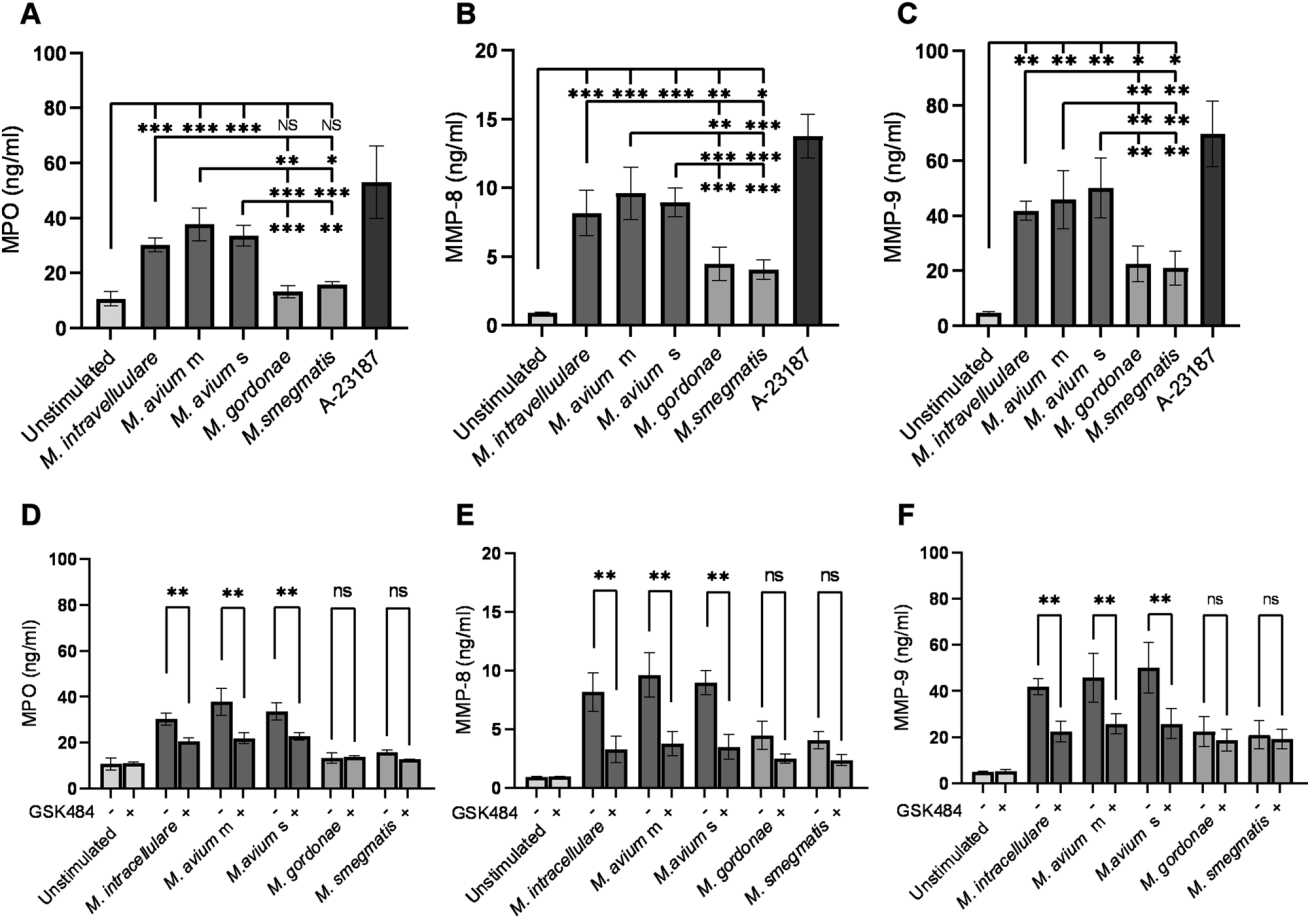
MAC-induced NET formation enhanced neutrophil degranulation. (**A**–**C**) Release of MPO (**A**), MMP-8 (**B**) and MMP-9 (**C**) from MAC-treated neutrophils. (**D**–**F**) Effect of PAD4 inhibitor on the release of MPO (**D**), MMP-8 (**E**) and MMP-9 (**F**) from MAC-treated neutrophils. MPO, MMP-8 and MMP-9 concentrations in cell culture supernatants were measured by ELISA. Data are means ± SD of three independent experiments. *P < 0.05, **P < 0.01, ***P < 0.001 by one-way analysis of variance (ANOVA) with Bonferroni's multiple comparison test.

NETs contain proteins from neutrophil granules and cytoplasm^17,40^, with their components being dependent on the stimulus^16^. Neutrophil granular enzymes, including MMP-8 and MMP-9, degrade extracellular matrix proteins and lead to lung tissue destruction^24^. The present study therefore investigated whether MAC induce the release of neutrophil granular enzymes during NET formation. MPO was detected in the culture supernatants of neutrophils treated with MAC but not non-pathogenic mycobacteria (Fig. 4A). MMP-8 and MMP-9 were released into the culture supernatants of all types of mycobacteria-treated neutrophils (Fig. 4B,C), with MAC inducing significantly greater MMP-8 and MMP-9 release than non-pathogenic mycobacteria. PAD4 inhibitor significantly reduced the release of MPO, MMP-8, and MMP-9 from MAC-treated neutrophils (Fig. 4D–F), but did not affect their release from neutrophils treated with non-pathogenic mycobacteria.

### MAC-induced IL-8 production by neutrophils

**Figure 5:**
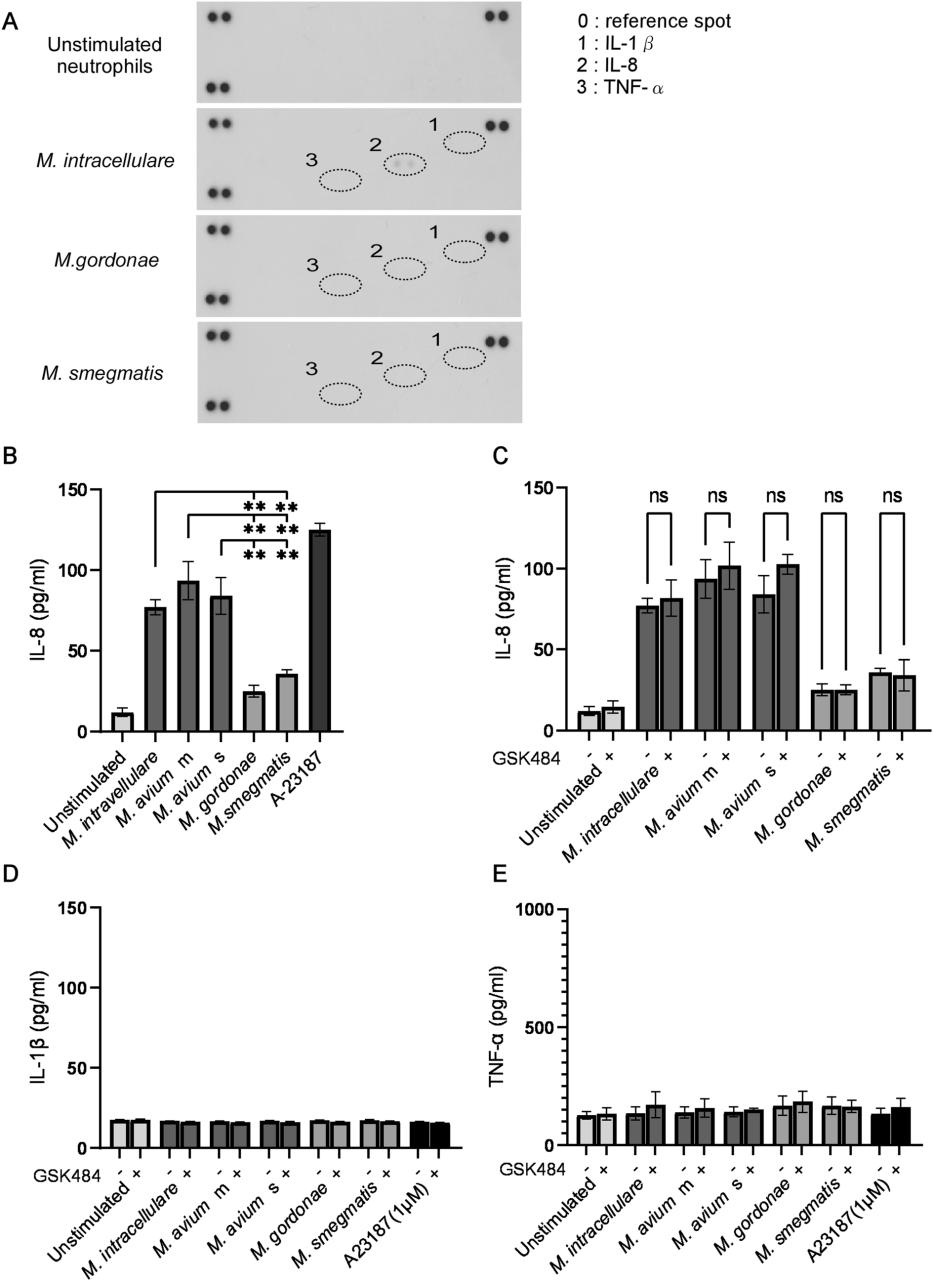
MAC induces IL-8 production by neutrophil. (**A**) Proteome profile of chemokines and cytokines in neutrophils treated with mycobacteria. Representative results are shown. (**B**) IL-8 production by neutrophils treated with mycobacteria. (**C**–**E**) Effects of PAD4 inhibitor on cytokine production by neutrophils treated with mycobacteria. The production of IL-8 (**B**,**C**), IL-1β (**D**) and TNF-α (**E**) by neutrophils was measured by ELISA. Data are means ± SD of three independent experiments. *P < 0.05, **P < 0.01, ***P < 0.001 by one-way analysis of variance (ANOVA) with Bonferroni’s multiple comparison test.

Human neutrophils secrete cytokines and chemokines in response to various stimuli, with these molecules regulating inflammatory and immune responses^41^. Assessment of cytokines and chemokines secreted by neutrophils treated with *M. intracellulare*, *M. gordonae* and *M. smegmatis* showed that, of the 36 cytokines and chemokines included in array analysis, only IL-8 secretion was observed (Fig. 5A). Because IL-1β and TNF-α are proinflammatory cytokines that promote neutrophilic inflammation^42^, the secretion of IL-8, IL-1β and TNF-α from mycobacteria-treated neutrophils was analyzed by ELISA. IL-8 was released from mycobacteria-treated neutrophils (Fig. 5B), with the amount secreted by MAC-treated neutrophils significantly higher than secreted by neutrophils treated with non-pathogenic mycobacteria. GSK484 did not affect the mycobacteria-induced IL-8 secretion by neutrophils (Fig. 5C). In agreement with the results of cytokine/chemokine array analysis, mycobacteria did not induce IL-1β or TNF-α release from neutrophils (Fig. 5D,E). These results indicate that the MAC-induced IL-8 production by neutrophils is not associated with NET formation.

## Discussion

NETs have been identified as the neutrophil preventing tool for microbial dissemination by trapping and killing bacteria^17^. However, the present study demonstrated that MAC, but not non-pathogenic mycobacteria, induced NET formation (Fig. 1C,D), but that MAC-induced NET formation was not involved in neutrophil killing of MAC (Fig. 3). *M. tuberculosis* was also demonstrated to escape from neutrophil killing regardless of NET formation^19^. Thus, NET formation is thought to be not involved in killings of pathogenic mycobacteria. In addition, *Streptococcus pneumoniae* and some strains of *Haemophilus influenzae*, which cause pneumonia, were found to escape from the bactericidal effects of NET formation^43,44^. Some pathogenic microorganisms that infect the lungs are likely to have avidity to avoid disinfection by NETs formation.

MAC and *M. tuberculosis* cause pathological changes in the lungs, such as bronchiectasis, granulation, and cavitation^45,46^. Bronchoalveolar lavage fluid (BALF) and plasma from patients with pulmonary tuberculosis contain increased levels of MMP-8 and MMP-9^47,48^. Besides, MMP-8 and MMP-9 were detected in lung tissue samples from patients with pulmonary tuberculosis^22^, and found to be associated with indications of lung pathogenesis, such as cavitation, granulation and bronchiectasis^28,49^. In this study, MAC induced MMP-8 and MMP-9 release from neutrophils, and these MMPs release were promoted via NET formation (Fig. 4). MMP-8 is capable of degrading collagen types I to III, which are constituents of the bronchi, blood vessels, pulmonary interstitium and cartilage, whereas MMP-9 mainly degrades collagen types IV and V, which are constituents of basement membrane^23^. Therefore, it seems that NET formation-promoted MMPs released from neutrophils cause pathological changes in the lungs of MAC infection.

MMP-8 and MMP-9 are released extracellularly as inactive forms and are subsequently activated at sites of inflammation^50^. The enzymatic activities of MMPs are suppressed by endogenous inhibitors, a class of molecules called tissue inhibitors of metalloproteinases (TIMPs), which bind active and inactive forms of MMPs^51^. MMP/TIMP imbalances have been associated with the progression of lung tissue destruction in tuberculosis^52^. The present study used MAC strains, including *M. avium*, isolated from patients with severe or mild lung tissue destruction. However, MAC strains from patients with severe and mild lung tissue destruction were similarly effective in inducing the release of MMPs (Fig. 4). Activation of MAC-induced MMPs may therefore be associated with the patient-dependent pathogenicity of MAC, although further studies are needed to understand the relationships between MAC pathogenicity and host susceptibility.

MAC patients with neutrophil predominance in BALF exhibit more severe CT imaging and greater progression than patients with lymphocyte predominance in BALF^53^. Although neutrophils produce a variety of cytokines and chemokines both in vivo and in vitro^41,54^, neutrophils produce fewer cytokines and chemokines per cell than monocytes/macrophages and lymphocytes^41^. Of the 36 cytokines assayed, only IL-8 was induced by MAC from neutrophils (Fig. 5A). IL-8, also known as CXCL8, is a CXC type proinflammatory chemokine that attracts and activates neutrophils^55^. Nuclear factor-kappa B (NF-κB) and c-Jun N-terminal kinase (JNK) have been identified as critical molecules for IL-8 transcription, translation, and expression^56^, but these cascades have not been associated with NET formation^57^. In consistent with those previous studies, PAD4 inhibitor had no effect on IL-8 production (Fig. 5C), although MAC-induced NET formation was mediated by PAD4 (Fig. 2). High concentrations of IL-8 (> 100 ng/mL) have been reported to induce NET formation by human neutrophils^17,58^. Moreover, isolated NETs can induce NET formation and IL-8 production by neutrophils^59^, suggesting MAC-induced NET formation and IL-8 production can coordinately induce additional neutrophil accumulation and activation at MAC-infected sites, exacerbating pathogenesis. Thus, neutrophils are likely to play detrimental roles in MAC infections via NET formation and IL-8 production.

Taken together, the present results clearly revealed that MAC not only escaped from killing by human neutrophils but also induced NET formation and the production of MMPs and IL-8. NET formation was not involved in MAC killing, suggesting that MAC strains utilize NET formation, release of MMPs and IL-8 production to promote the progression of lung infections. These three processes may therefore be therapeutic targets in patients with MAC infection. Further study of the molecular mechanisms of MAC induced neutrophil activation is needed to elucidate the pathogenesis of MAC infections.

## Supplementary Information

Supplementary Information.
