# Supplementary material for: Mycobacterium avium-intracellulare complex promote release of pro-inflammatory enzymes matrix metalloproteinases by inducing neutrophil extracellular trap formation
Source: Sci Rep. 2022 Apr 11;12:5181. doi: 10.1038/s41598-022-09017-y (PMC9001666; doi:10.1038/s41598-022-09017-y)
Supplement: Supplementary file 1 — Supplementary Information. [file 41598_2022_9017_MOESM1_ESM.pdf]

Supplementary Figure 1

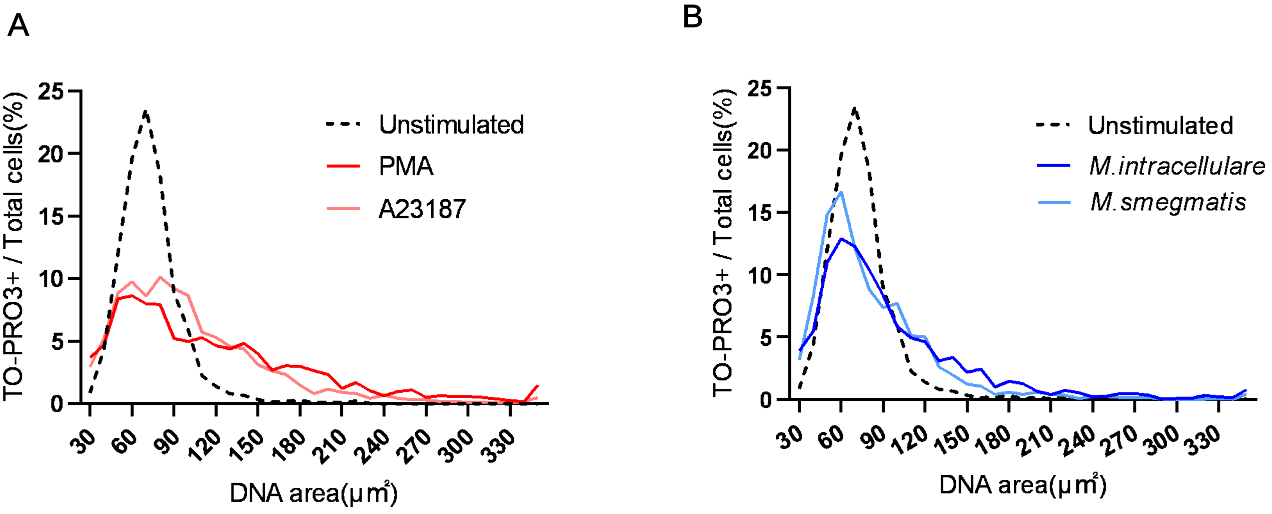

Figure S1 Association of the expansion of the nuclear area of neutrophils with NET formation. (A) Size distributions of the nuclear areas of unstimulated neutrophils (black dotted line) and neutrophils stimulated with PMA (red) and A-23187 (pink). (B) Size distributions of the nuclear areas of unstimulated neutrophils (black dotted line) and neutrophils treated with M. intracellulare (MAC) (blue) and M. smegmatis (light blue). 300 cells per treatment were analyzed.

Supplementary Figure 2

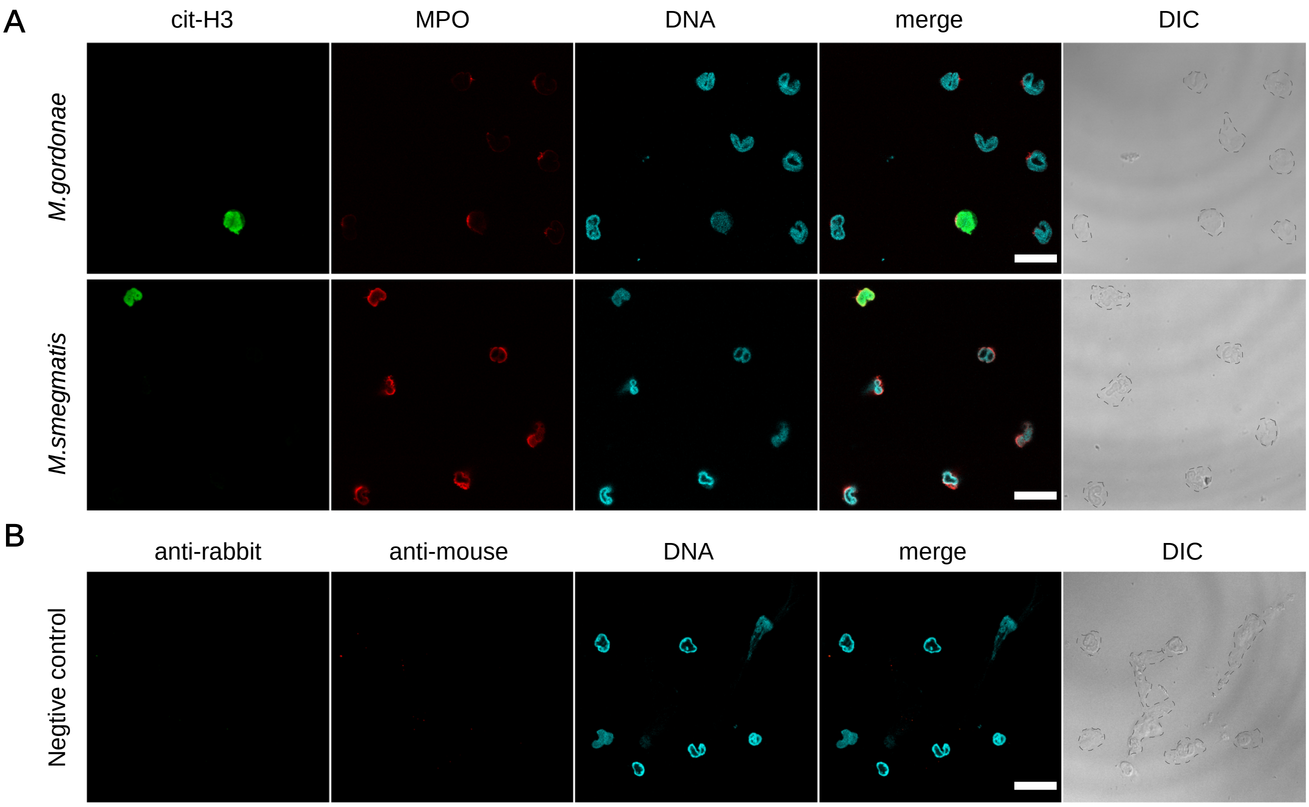

Figure S2 Non-pathogenic mycobacteria have little effect on NET formation. (A) Confocal images of non-pathogenic mycobacteria-induced NET formation. Neutrophils were stained to identify nuclei (blue) and areas positive for cit-H3 (green) and MPO (red) to determine the structures of NETs. (B) Confocal images of negative antibody controls used for immunofluorescent staining. Neutrophils were treated with M. intracellulare and stained with anti-rabbit antibody (green) and anti-mouse antibody (red). Gray dotted lines in DIC images indicating the outline of neutrophils. Confocal images were acquired on a Leica TCS-SP5 confocal microscope equipped with a 1.4 numerical aperture (NA) ×63 objective lens. DIC; differential interference contrast. white scale bar; 25 μm.

Supplementary Figure 3

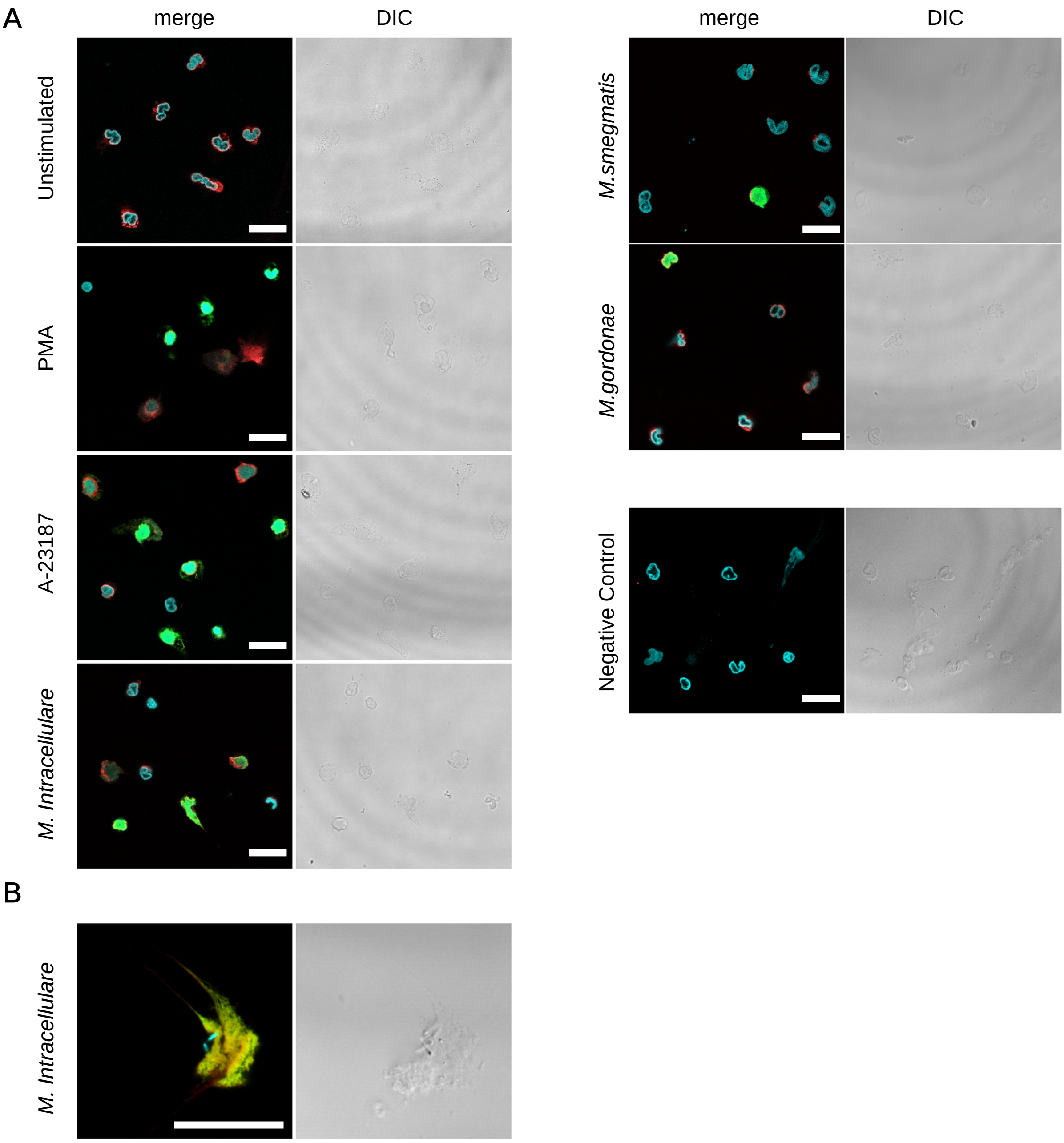

Figure S3 Original DIC images of immunofluorescence staining. (A, B) Confocal images were acquired on a Leica TCS-SP5 confocal microscope equipped with a 1.4 numerical aperture (NA) ×63 objective lens. DIC; differential interference contrast. white scale bar; 25 μm .

Supplementary Figure 4

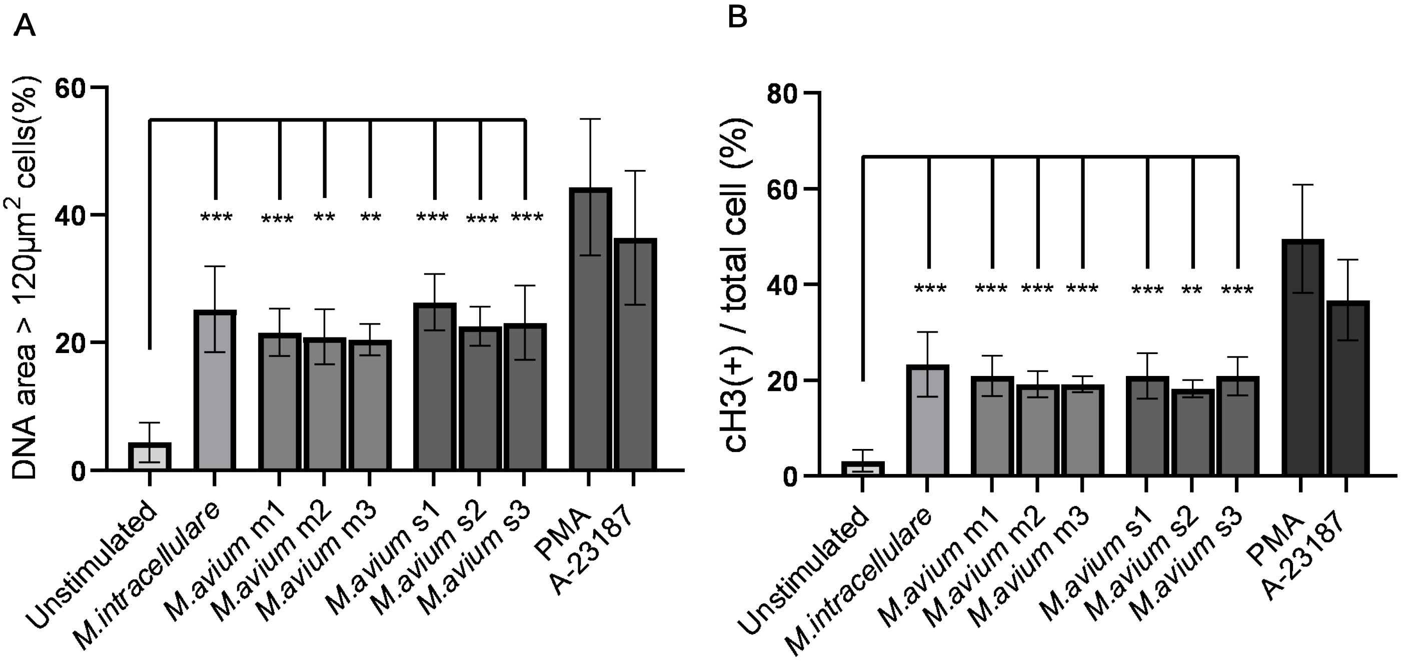

Figure S4 Effect of MAC from patients with severe or mild lung tissue distraction on NET formation. (A, B) Assessment of NET formation by DNA area expansion based on the percentage of cells with a nuclear area > 120 μm2 (A) and the percentage of cit-H3 positive neutrophils (B). M. avium m1, m2 and m3 were the mycobacteria isolated from different patients with mild lung tissue destruction and a favorable clinical course, while M. avium s1, s2 and s3 were the mycobacteria isolated from different patients with severe lung tissue destruction and a critical clinical course. Data shown are the means ± standard deviations (SD) of three independent experiments. *P<0.05, **P<0.01, ***P<0.001 by one-way analysis of variance (ANOVA) with Bonferroni's multiple comparison test.

Supplementary Figure 5

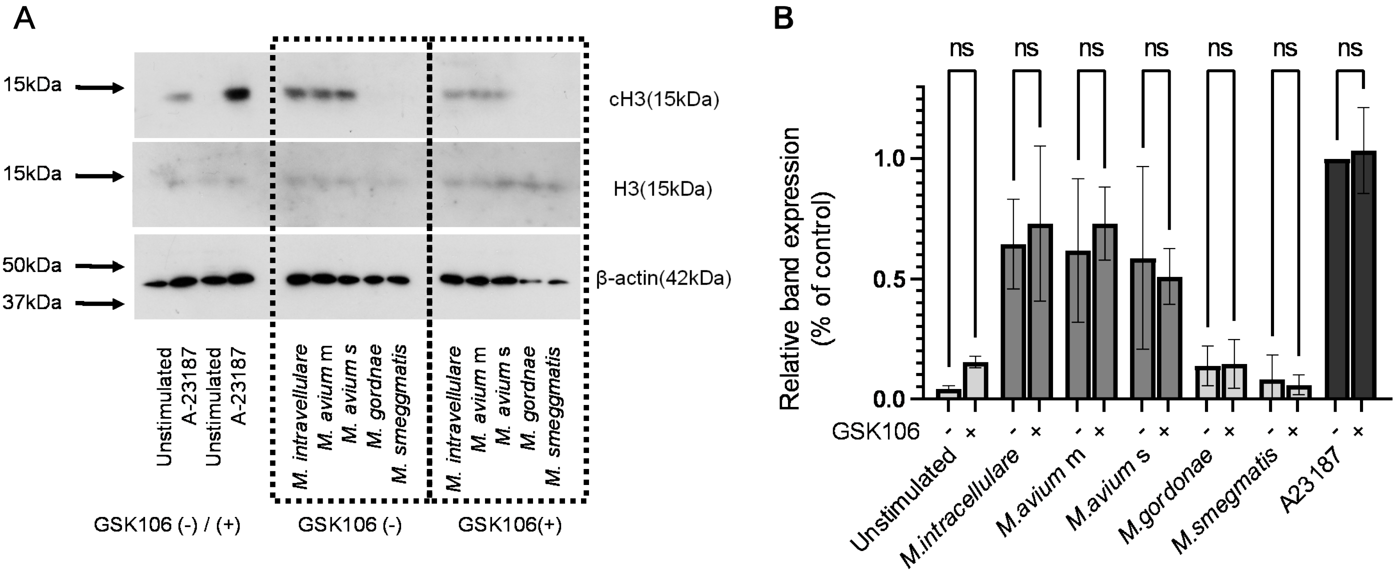

Figure S5 GSK106 does not affect NET formation. (A) Effect of GSK106, the negative control for GSK484, on the levels of cit-H3 expression. Representative protein bands are shown. Molecular weights (kDa) are indicated on the left. H3 and β-actin were used as an internal controls. Blots were cropped from different parts of the same gel. (B) Relative band intensities were calculated on the basis of densitometric analysis. Original blots are presented in Supplementary Fig. 6.

Supplementary Figure 6

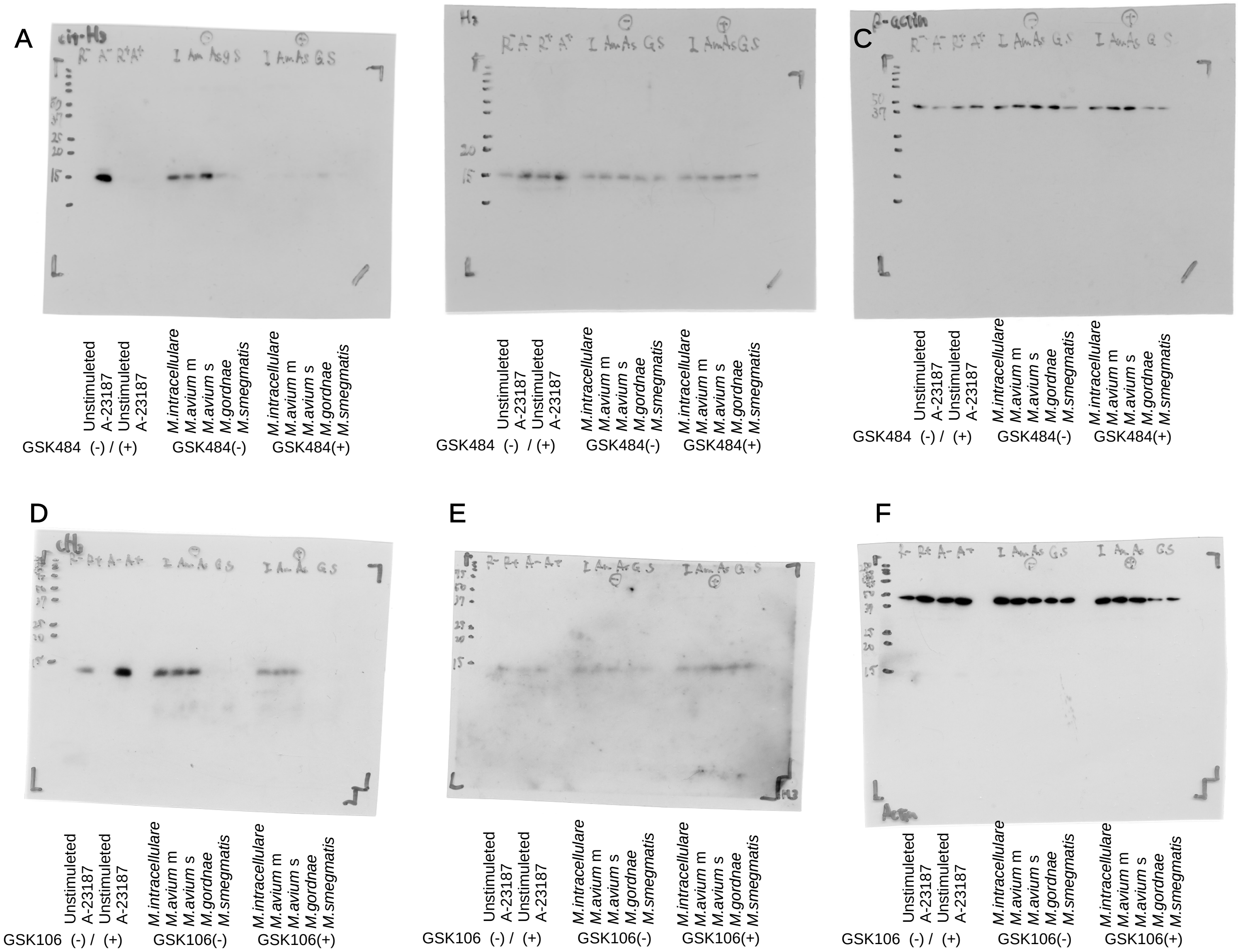

Figure S6 Original western blots including protein ladders of data depicted in Figure 3(A,B,C) and Supplementary Figure 5(D,E,F). SDS PAGE was performed and proteins were transferred onto a PVDF membrane by a semi-dry process. Membranes were probed with appropriate primary and secondary antibodies (for respective antibodies see “Materials and Methods”) conjugated with horseradish peroxidase. Molecular weights (kDa) are indicated on the left. (A,D)cit-H3, (B,E) H3, (C,F) β-actin.
